# Nutrition and *Helicobacter pylori*: Host Diet and Nutritional Immunity Influence Bacterial Virulence and Disease Outcome

**DOI:** 10.1155/2016/3019362

**Published:** 2016-09-05

**Authors:** Kathryn P. Haley, Jennifer A. Gaddy

**Affiliations:** ^1^Department of Medicine, Vanderbilt University School of Medicine, Nashville, TN, USA; ^2^Veterans Affairs Tennessee Valley Healthcare Services, Nashville, TN, USA

## Abstract

*Helicobacter pylori* colonizes the stomachs of greater than 50% of the world's human population making it arguably one of the most successful bacterial pathogens. Chronic* H. pylori* colonization results in gastritis in nearly all patients; however in a subset of people, persistent infection with* H. pylori* is associated with an increased risk for more severe disease outcomes including B-cell lymphoma of mucosal-associated lymphoid tissue (MALT lymphoma) and invasive adenocarcinoma. Research aimed at elucidating determinants that mediate disease progression has revealed genetic differences in both humans and* H. pylori* which increase the risk for developing gastric cancer. Furthermore, host diet and nutrition status have been shown to influence* H. pylori*-associated disease outcomes. In this review we will discuss how* H. pylori* is able to create a replicative niche within the hostile host environment by subverting and modifying the host-generated immune response as well as successfully competing for limited nutrients such as transition metals by deploying an arsenal of metal acquisition proteins and virulence factors. Lastly, we will discuss how micronutrient availability or alterations in the gastric microbiome may exacerbate negative disease outcomes associated with* H. pylori* colonization.

## 1. *H. pylori* Infects the Human Stomach

*Helicobacter pylori* is a Gram-negative member of the Epsilonproteobacteria class. Over 50% of the global human population is colonized with* H. pylori*, which inhabits the gastric niche of human hosts and is commonly acquired early in life. Furthermore, evidence indicates that* H. pylori* has colonized human hosts and coevolved for at least a thousand centuries [1–4]. The human stomach provides numerous nutritional opportunities and challenges for an invading prokaryote. To colonize the stomach successfully,* H. pylori* must survive the acidic pH in the lumen of the stomach, move through the mucus lining of the gastric tissue via chemotactic flagellar-mediated motility, attach to gastric epithelial cells using a repertoire of adhesins, and deploy cytotoxins to alter the gastric environment and create a hospitable niche for bacterial proliferation [3]. These bacterial toxins promote necrosis, autophagy, and proinflammatory signaling cascades [4, 5]. However,* H. pylori* persists in the stomach despite a robust inflammatory response, indicating that this organism has evolved elaborate mechanisms to circumnavigate the onslaught of host immunity [4–6].

## 2. *H. pylori* Infection and Disease Outcomes

Virtually all hosts infected with* H. pylori* experience gastritis while a smaller subset of these patients develop more serious outcomes such as peptic or duodenal ulcer, MALT lymphoma, or gastric adenocarcinoma. Nearly 75% of all gastric cancer and 5.5% of all malignancies worldwide can be attributed to* H. pylori* [4].* H. pylori* infection is the strongest risk factor for developing gastric cancer [5]. It is proposed that the profound proinflammatory signaling initiated by* H. pylori* infection leads to atrophic gastritis, intestinal metaplasia, dysplasia, and finally gastric cancer [6]. This process, termed the “Correa pathway” is predicated on the chronic inflammation of the gastric mucosa which fosters a cascade of genotypic perturbations that ultimately lead to carcinogenesis [6–9]. It is increasingly appreciated that carcinogenesis is established due to a constellation of factors including host genetics, environment, and bacterial strain differences [6–10]. A better understanding of how these factors intersect to promote disease progression could yield novel preventative or therapeutic strategies to ameliorate the global disease burden, which costs hundreds of thousands of human lives each year [10]. In this review we consider how nutrition, or the process by which an organism derives cofactors and metabolic precursors, impacts the progression of* H. pylori*-associated disease outcomes and gastric homeostasis. Furthermore, we discuss how host micronutrients can alter bacterial growth and virulence and ultimately influence pathogenesis.

*H. pylori* has an ancient association with human beings [1]. Although* H. pylori* strains exhibit remarkable genetic diversity, phylogenetic analyses have revealed that strains can be classified into distinct phylogeographic clades indicative of their origin [2, 3]. These results indicate that* H. pylori* strains have coevolved with their hosts, observations which are supported by results indicating that* H. pylori* has undergone reductive evolution during its association with man [11]. However, prolonged coevolution is commonly associated with commensal adaptation and concurrent loss of virulence [12, 13]. Because* H. pylori* exhibits strain-specific virulence and potential to cause disease, this supports a model in which the coevolution of* H. pylori* and its cognate human host has been perturbed [2, 3].

In some geographical settings, such as Asia,* H. pylori* infection and gastric cancer rates are correlative. However, in other areas, such as Africa, Malaysia, India, and Costa Rica, infection rates are high and gastric cancer rates are low [14–17]. These are collectively referred to as “enigmas” because the protective mechanisms in these populations are obscure. It is proposed that* H. pylori* potentially coevolves with its host to dampen pathogenic effects and promote immunological tolerance which facilitates protection against numerous autoimmune diseases including allergic airway disease [18, 19]. However, the role of geography, nutrition, and host genetics remains ill-defined in this model. Furthermore, regions within a single country, such as Colombia, experience differential disease outcomes [20]. Recent assessments of genetic variations in both host and* H. pylori* strain by multilocus sequence typing analyses (MLST) were performed to ascertain how the coevolutionary relationships between hosts and pathogens were shaping development of gastric cancer [2]. This work demonstrated that low-risk coastal Colombians exhibit phylogenetic variations consistent with an admixture of Amerindian, European, and African populations. Similarly,* H. pylori* strains recovered from these individuals primarily represented an African lineage of* H. pylori* that was concordant with the host genetic background [2, 3]. Conversely, mountain-dwelling Colombians exhibit phylogenetic variations consistent with Amerindian heritage and their* H. pylori* strains predominantly were associated with a European phylogenetic clade [2, 3]. The authors conclude that infection with a strain of* H. pylori* that is discordant with host phylogenetic background is predictive for increased risk of gastric cancer [2].

## 3. *H. pylori* Virulence Factors

Besides phylogenetic differences between host and pathogen, there are specific strain differences that have been associated with increased risk of gastric disease.* H. pylori* strains that harbor a 40 kb genomic island termed the “*cag*-pathogenicity island” (*cag*-PAI) have been associated with increased risk of gastric disease outcome [21]. The* cag*-PAI encodes a type IV secretion system (*cag*-T4SS) which is a macromolecular nanomachine that spans both the inner and outer membrane of* H. pylori*. The* cag*-T4SS functions to transport substrates, such as peptidoglycan, and effector molecules, such as the oncogenic cytotoxin CagA, from the bacterial cytoplasm into the host epithelial cell. The activity of the T4SS has multiple effects on the host including nuclear factor *κ*B activation, IL-8 chemokine secretion, host cytoskeletal rearrangement, and recruitment of innate immune cells to the site of infection [22–25]. In addition to the* cag*-T4SS cytotoxin secretion,* H. pylori* also secretes a pore-forming cytotoxin, VacA [26]. VacA is an 88-kDa protein that is secreted through type V, or autotransporter secretion pathway [27]. It causes a variety of alterations in target cells including vacuolation, depolarization of membrane potential, permeabilization, disruption of endosomal and lysosomal trafficking, autophagy, programmed necrosis, and immune modulation including inhibition of T cell activation and proliferation. Interestingly, VacA and CagA appear to have antagonistic properties: CagA is highly proinflammatory, while VacA is immunosuppressive, and VacA induces CagA degradation via autophagic pathways [22, 27, 28]. Interestingly, both VacA and CagA are often coregulated in response to nutritional signals, indicating that* H. pylori* has evolved to utilize both of these toxins in concert under certain nutritional stresses [29]. Together, these two cytotoxins promote* H. pylori*-dependent pathogenesis.

Additionally,* H. pylori* utilizes a repertoire of outer membrane proteins to facilitate host-pathogen interactions. The adhesin BabA binds mucosal ABO/Lewis-B blood group carbohydrates and consequently facilitates adhesion to gastric surfaces. Adherence to the gastric mucosa and/or epithelial surface is a critical first step in colonization and ultimately aids bacterial virulence by promoting the interaction of the* cag*-T4SS with host cells [30, 31]. Another adhesin, SabA, binds to laminin and sialyl-dimaric-Lewis × glycosphingolipid receptor and is a member of the BabA protein family [32]. Upon binding to the receptor, SabA promotes hemagglutination via sialyl-Lex binding, a process that is critical for survival within the hostile gastric environment [33]. Additionally,* H. pylori* outer membrane protein and Hop-family proteins such as outer membrane inflammatory protein A (OipA, encoded by* hopH*) or HopZ protein are both required for gastric epithelial cell binding [33]. Although the host receptors for these proteins have not yet been identified, both proteins have been implicated in inflammation and/or carcinogenesis [34, 35]. Interestingly, there is a high degree of variation in the sequence of CagA, VacA, BabA, SabA, OipA, and HopZ, indicating that* H. pylori* adapts to its host by modifying the repertoire of virulence factors to accommodate niche-specific challenges [36].

## 4. *H. pylori* and Nutrition

In addition to host or strain genetic differences, environmental factors, such as host diet, are emerging as important components of the ecology within the gastric environment. It is likely that the gastric environment is highly influenced by host nutrient intake. Epidemiological studies have revealed that dietary habits such as high intake of green tea, fruits, or vegetables are protective against gastric cancer risk [37–39]. Conversely, case-controlled and cohort studies reveal that high intake of red meat and/or processed meat (which are high in transition metals) and preserved foods (pickled, dried, smoked, or salted) which are often high in salt is associated with increased risk of noncardia gastric cancer [40, 41]. Furthermore, the advent of refrigeration has radically changed the manner in which food is prepared for storage. Case-controlled population studies have demonstrated that access to refrigeration is protective against gastric cancer [42]. This is attributed to the fact that refrigeration leads to prolonged access to fresh foods such as fruits and vegetables, which would otherwise be unavailable. It is hypothesized that carotenoids, folate, vitamin C, and phytochemicals from fruits and vegetables have a protective role against carcinogenesis. Conversely, salt and the availability of some transition metals can alter* H. pylori* virulence and accelerate carcinogenesis [43, 44]. The contribution of these individual micronutrients to* H. pylori*-dependent diseases will be reviewed in detail below.

### 4.1. Salt

Gastric cancer is the third leading cause of death from cancer worldwide. While large geographic and ethnic differences in gastric cancer incidence exist, a common risk factor for gastric cancer development is high levels of dietary salt intake. A meta-analysis of studies analysing the association between diets rich in salt and gastric cancer risk concluded that salt consumption is directly associated with the risk of gastric cancer [45]. Furthermore, the risk of developing cancer increases with increased salt ingestion in a dose-dependent manner [46]. Studies included in this meta-analysis looked at the association between high salt diets and gastric cancer across a spectrum of countries and ethnicities. For example, the meta-analysis included studies which found a correlation between consumption of salty foods, such as miso soup, pickled vegetables, and salted fish within Japanese people, and a study conducted in Norway evaluating the risk of total salt intake and gastric carcinoma. Also included in this meta-analysis are studies which show no correlation between excessively salted foods and cancer; however the strain of* H. pylori* endemic to these regions lacks* cagA* and is associated with a decreased risk of gastric cancer as compared to strains harboring* cagA*. Additional studies indicated that the association between salt consumption and gastric cancer risk was highest amongst individuals who were habitual consumers of high salt foods [45]. The rationale for this association between heavy salt intake and gastric cancer is multifaceted and includes that salt perturbs the integrity and viscosity of gastric mucosa and promotes colonization by* H. pylori* both of which ultimately contribute to increased inflammation and subsequent gastric cell proliferation and endogenous DNA mutations [47–49]. One such study compared gastric tissue morphology of mice maintained on a standard diet compared to mice sustained on a high salt diet and found that animals within the high salt cohort had increased gastric epithelial cell hyperplasia and concomitant loss of parietal cells [49].

While high levels of salt consumption in the absence of* H. pylori* infection are associated with gastric cancer, the alterations to the gastric tissue mediated by high salt intake are further exacerbated by* H. pylori* colonization and drive disease progression. Studies aimed at elucidating the molecular mechanisms responsible for this increased susceptibility to cancer development have revealed a complex relationship whereby increased salt ingestion potentiates* H. pylori* carcinogenesis. In addition to promoting* H. pylori* colonization of the gastric mucosa high dietary salt exacerbates* H. pylori* induced inflammation. Studies performed in a Mongolian gerbil model determined that* H. pylori* infected animals maintained on a high salt diet had increased inflammation when compared to infected animals maintained on a normal diet [50]. The increase in inflammation was assessed using both histological examination of gastric tissue and comparing levels of the proinflammatory cytokine, IL-1*β*. Importantly, this increase in inflammation mediated by high salt levels was CagA dependent, and animals infected with a* cagA* deficient strain of* H. pylori* had significantly less inflammation, even in the context of high salt [50]. Studies investigating the regulation of* cagA* have found that its expression is increased in response to multiple environmental changes including increases in environmental salt concentrations [51]. In fact, this increase in* cagA* expression was detected* in vivo*, using RT-PCR on gastric tissue samples from infected animals. Accompanying the increase in inflammation, infected animals on a high salt chow were found to have augmented dysplasia and invasive gastric adenocarcinoma [50]. Concomitant with this disruption in tissue architecture and inflammation is an increase in* H. pylori* induced hypochlorydia in animals fed excessive salt [50]. Alterations in salt concentration also enhance production of several* H. pylori* outer membrane proteins notably, including HopQ, which is upregulated in response to high salt stress, and VacA which is upregulated in low salt conditions [52]. Together these studies indicate that increases in salt consumption result in alterations to both the host and* H. pylori* and this constellation of changes stimulates carcinogenesis.

### 4.2. Iron

Iron is an essential nutrient for nearly every living organism including* H. pylori* [53]. Iron is frequently used as an enzymatic cofactor and plays a critical role in respiration and electron transport [54]. To prevent bacterial growth, the human body exploits this need for iron by limiting bacterial access to this vital metal and sequestering iron intracellularly in a process referred to as nutritional immunity [55]. The majority of iron within the human body is localized within erythrocytes in the form of heme, a tetrapyrrole ring with a coordinated iron center. Heme is then further complexed within hemoglobin [56]. Any extracellular iron is rapidly removed by high-affinity iron binding proteins such as lactoferrin and transferrin [57]. Nutritional immunity is a dynamic process capable of responding to pathogenic assaults on the host. Iron absorption and distribution are regulated through the hepatic peptide hormone, hepcidin. During the infectious process, inflammation can mediate increases in hepcidin leading to a hypoferremic response that depletes even further the available iron present within the host [58]. Together lactoferrin, transferrin, hepcidin, and numerous other proteins ensure that the human body has an inhospitably low level of iron available to invading bacteria.

While the human stomach is a unique organ in that it experiences large influxes of iron during digestion, the specific niche occupied by* H. pylori* is within the gastric mucosa, an area predicted to have little available iron [59].* H. pylori* has evolved sophisticated mechanisms to circumvent the host's sequestration of iron and responds to the scarcity of this metal with a coordinated upregulation of iron acquisition systems and virulence factors [59–66]. One way that* H. pylori* mediates gene regulation in response to low environmental iron levels is through the global ferric uptake regulator (Fur), a transcriptional regulator [61–63].* H. pylori* Fur is unique in that it can bind DNA sequences both when complexed to ferric iron and in its apo form [60]. Consequently,* H. pylori* Fur can regulate gene expression in response to conditions of both high and low iron. Many of the Fur regulated genes that are transcriptionally upregulated upon iron starvation facilitate the acquisition and trafficking of iron within the bacterial cell [60–62]. For example, when iron availability is low* H. pylori* increases the transcription of the high-affinity iron transporters* feA1, fecA2, frpB1*, and* feoB* facilitating an influx of iron into the cytoplasm [60–65]. Additionally,* H. pylori* increases binding of the host chelating proteins, lactoferrin and transferrin, upon iron starvation, both of which can be used as a source of nutrient iron. This increase in lactoferrin and transferrin binding is presumably through increasing transcription of the receptors for these proteins [59]. Together this coordinated upregulation of iron acquisition genes allows* H. pylori* to respond to and survive the iron deplete environment of the human host.

Many pathogenic bacteria coordinate the expression of virulence factors to the detection of changes in iron availability and* H. pylori* is no exception. Two of the most important virulence factors expressed by* H. pylori*, VacA and CagA toxin, are transcriptionally regulated in part by iron [63, 64]. Similar to VacA, once inside the cytoplasm, CagA mediates a cascade of changes within the cell including changes to cell morphology and immune signaling. Importantly, the activity of CagA in concert with VacA has been shown to initiate a perturbation in the inner leaflet of the cell membrane which results in the rerouting of transferrin receptors to the apical surface, ostensibly making all bound transferrin available to the bacterium [67]. Similarly, the human antimicrobial protein lactoferrin, which serves as an iron source for* H. pylori*, has been shown to repress the expression of both* cagA* and* vacA*, indicating that the human antimicrobial response can directly alter* H. pylori* virulence by altering the micronutrient gradient available to this bacterial pathogen [59, 68]. Recently, our work has indicated that the biogenesis and activity of the* cag-*T4SS increase upon iron starvation [65, 66]. Together these findings indicate that regulation of* H. pylori* toxin secretion is mediated by iron availability and that both toxins play a critical role in iron homeostasis.

Iron availability not only modulates expression and deployment of both* vacA* and* cagA-*T4SS* in vitro* but recent research utilizing a Mongolian gerbil infection model indicates that dietary iron levels augment disease progression and cancer development* in vivo*. In this infection model gerbils were maintained on iron replete and iron deplete diets beginning two weeks prior to infection and were maintained on these diets throughout the duration of the infection. Analysis of animals treated with a low iron diet revealed that they had markedly less hepatic iron present, as well as significantly less iron binding proteins, ferritin and hemoglobin, within their serum [66]. Iron levels within gastric tissue were also measured using Inductively Coupled-Plasma Mass-Spectrometry (ICP-MS) which demonstrated that iron concentrations within the replicative niche of* H. pylori* were drastically reduced upon subjection to an iron deplete diet [66]. Together these results confirm that an iron poor diet results in a global decrease in iron stores throughout the body, including the stomach. Within the same study, when comparing the disease outcome of animals fed an iron deficient diet to that of animals maintained on an iron sufficient diet, it was clear that the animals with decreased iron had greater immune cell infiltrate to the site of infection, a more rapid onset of gastritis, and a higher rate of cancer development, compared to the animals maintained on an iron rich diet [66]. The mechanisms driving these differences in inflammation and cancer development were found to be similar* in vivo* as they are* in vitro*, in the fact that the increased inflammation and disease severity are attributable to the deployment of the* cag*T4SS pili [65, 66]. The number of pili found in animals maintained on low iron diets versus high iron diets was determined using SEM to visualize and subsequently enumerate the amount of pili formation under both conditions. Consistent with this finding, strains harvested from iron deplete animals translocated a greater amount of CagA into host cells than strains from animals fed an iron rich diet. Together these data demonstrate that the diet of the host, specifically iron intake, has a large impact on the availability of nutrients for invading pathogens and consequently influences disease outcomes.

The correlation between reduced dietary iron and increased disease severity demonstrated in an animal model is mirrored within the human population. Individuals with low serum levels of the iron binding protein, ferritin, have more severe disease outcomes in the context of an* H. pylori* infection than individuals with adequate ferritin serum levels [69]. The mechanisms by which iron deficiency can arise are varied and include not only diets lacking necessary iron but also blood loss. Some strains of* H. pylori* are associated with hemorrhagic gastritis which may contribute to blood loss and successive iron deficiency. Furthermore, chronic* H. pylori* infection is associated with hypochlorhydria, an increased stomach pH, which may impede iron absorption as iron is more soluble at lower pHs. Importantly, case control studies have shown an inverse relationship between dietary iron intake and gastric cancer suggesting that iron deficiency arising from both dietary factors and blood loss contributes to cancer progression during an* H. pylori* infection [70, 71].

### 4.3. Zinc

Similar to iron, zinc is gaining appreciation as a micronutrient that exerts great influence at the host-pathogen interface. Zinc is required for cellular processes in all domains of life, and the mammalian host exploits this requirement by chelating nutrient zinc within host innate immune S100A-family proteins, including EN-RAGE (calgranulin C, S100A12) or calprotectin (MRP-8, S100A8/A9) [72, 73]. This process, termed “nutritional immunity,” tightly regulates zinc availability in response to infection and essentially starves the invading prokaryote. Both EN-RAGE and calprotectin are significantly elevated in* H. pylori* infected gastric tissues compared to uninfected tissues, and these proteins primarily localize to polymorphonuclear cells (neutrophils) recruited to the site of infection [72, 73]. The severity of inflammation, specifically the infiltration of neutrophils in response to* H. pylori* infection, was inversely proportional to mucosal zinc levels [74]. The authors conclude that low zinc levels could enhance inflammation, but it is equally plausible that the S100A-family proteins deposited by neutrophils at the site of infection could be contributing to the chelation and subsequent removal of zinc from the gastric mucosa.

*H. pylori* has a strict nutritional requirement for zinc to grow and both calprotectin and EN-RAGE have been shown to inhibit* H. pylori* growth and viability via zinc sequestration activity [72, 73]. In response to zinc sequestration (by calprotectin or synthetic chelators),* H. pylori* forms tenacious biofilms and alters its lipid A structure [75]. The alterations in lipid A structure under conditions of zinc starvation indicate that LpxF, LpxL, and LpxR enzyme functions are diminished [75]. This results in the presence of a lipid A structure which is penta-acylated and contains both a phosphoethanolamine residue at 1′-position and a 4′-phosphate which decorates the outer membrane [75]. These alterations in lipid A structure confer decreased cell surface hydrophobicity which enhances bacterial fitness in the presence of calprotectin. These results indicate that* H. pylori* modifies its lipopolysaccharide endotoxin production in response to nutrient zinc availability to circumnavigate the host immune response [75].

Interestingly,* H. pylori* exposure to EN-RAGE or calprotectin prior to coculture with gastric epithelial cells also results in diminished* cag*-T4SS activity including CagA translocation into host cells and proinflammatory IL-8 chemokine secretion. Additionally, the downregulation of* cag*-T4SS activity is associated with abrogation of* cag*-T4SS pilus deployment, results that were reversed by the addition of an exogenous source of nutrient zinc [65, 72, 73]. Together, these results indicate that* H. pylori* senses nutrient zinc in the gastric environment and has evolved to deploy the* cag*-T4SS in response to the presence of this transition metal. Epidemiological data supports a model in which zinc enhances the carcinogenic* cag*-T4SS activity, as high zinc intake has been associated with gastric noncardia adenocarcinoma [76]. Concordantly, high serum zinc levels and high zinc intake have been associated with* H. pylori* infection and antibody response, respectively [77]. Similar studies in pediatric patients have revealed no association between* H. pylori* and iron or zinc nutritional status but significant association between* H. pylori* infection status and copper nutritional status as determined by serum metal concentrations [78, 79].

Besides regulating endotoxin and cytotoxin secretion, zinc has also been implicated as an important cofactor for urease and nickel-iron hydrogenase (Ni, Fe-hydrogenase), enzymes that are critical for* H. pylori* survival in the low pH of the stomach [80–82]. Zinc is required for dimerization of the chaperone UreG, which participates in nickel trafficking to promote urease activation. The accessory protein UreE utilizes either nickel or zinc as a cofactor which is critical for activity [83]. The metallochaperone, HypA, binds zinc for appropriate structural stabilization and interacts with HypB to deliver nickel to both urease and Ni, Fe-hydrogenase, indicating that zinc is critical for bacterial physiology* in vivo* [84–86].

*H. pylori* has clearly evolved to experience zinc stress in the gastrointestinal environment due to evidence that this pathogen encodes multiple proteins involved in zinc efflux in its genome. CadA, CznABC, CrdB, and CzcAB proteins protect* H. pylori* from zinc toxicity [87–89]. CznABC efflux function is required for colonization in a gerbil model of* H. pylori* infection. These studies underscore the critical role that detoxification strategies play in bacterial metal homeostasis during pathogenesis. It is likely that* H. pylori* encounters transition metals including zinc in the micromolar range from the host diet [89]. Studies on short term supplementation with zinc sulfate reveal that cohorts maintained on zinc supplementation exhibit less gastritis than cohorts without zinc supplementation. However, bacterial burden was not altered by this addition of zinc, as would be expected with increased zinc toxicity within the bacterial cell [90]. The numerous epidemiological studies of dietary zinc intake and zinc supplementation have yielded heterogeneous results, and a recent meta-analysis concluded that no firm conclusions about dietary zinc could be reached at this time [91]. It is interesting to note that, of the studies which have shown a correlation between zinc intake and* H. pylori*-dependent disease progression, most have been on populations in Asia, which are commonly associated with* cag*-PAI-positive strains of* H. pylori* [16].

### 4.4. Nickel

In addition to zinc and iron,* H. pylori* requires the transition metal nickel for full virulence.* H. pylori* exploits nickel-containing metalloenzymes such as NiFe-hydrogenase and urease to circumnavigate the low pH environment of the human stomach [92]. Urease, one of the most abundant enzymes in* H. pylori* proteome requires 24 nickel atoms for activity [93]. However, excess nickel in the bacterial cell results in mismetallation of cellular enzymes, which can abrogate physiological activity. Consequently,* H. pylori* manages its cellular nickel economy by tightly controlling both nickel import and export functions [94]. Nickel is transported into the bacterial cell in* Helicobacter* spp. via a NixA permease, and the FrpB4 outer membrane protein in a TonB-dependent fashion [95–98]. Nickel efflux is achieved by the promiscuous CznABC transporter, which promotes nickel resistance in* H. pylori* cells [89]. It is likely that* H. pylori* will encounter micromolar concentrations of transition metals such as nickel from host diet, which could ultimately influence the activity of these critical enzymes and nickel homeostasis functions [89]. Recent work by Campanale et al. indicates maintenance of patients on a nickel-free diet enhanced* H. pylori* eradication rate, supporting the essentiality of nickel for* H. pylori* pathogenesis [92].

## 5. Microbiome

Chronic* H. pylori* colonization leads to dramatic changes within the gastric environment including a reduction in parietal cells and subsequent increases in stomach pH and altered nutrient availability and local immune responses. Together, these* H. pylori* mediated changes in gastric physiology and immunology likely induces perturbations in the microbiome composition. While* H. pylori*-associated changes in microbiome structure are not fully understood, recent advances in both DNA sequencing and computational analysis have revealed an exceptionally complex microbiota in the human stomach.* H. pylori* colonization in specific pathogen-free female BALB/c mice leads to a decrease in the quantity of* Lactobacillus* species within the gastric microbiota when compared to noninfected mice [99]. In contrast* H. pylori* infection did not significantly alter the overall stomach microbiota composition within female C57BL/6N mice. Infection models using Mongolian gerbils found that* H. pylori* colonization altered both the number and localization of indigenous gastric microbiota ultimately leading to more severe gastritis [100]. Analysis of the microflora following a 12-week infection found dramatic differences in composition including the appearance of* S. aureus* and Enterococci and a decrease in number of Lactobacilli as well as an increase in number of* Bacteroides* [100]. Furthermore, gerbil studies have shown that* H. pylori* infected animals had alterations in the distribution of Bifidobacteria which was greater in the corpus than the antrum when compared to uninfected animals. Similarly, Aebischer et al. found that the stomachs from* H. pylori*-positive animals were colonized by bacterial species typically confined to the lower gastrointestinal tract [99]. Underscoring the complex relationship that exists between* H. pylori* and the indigenous microflora are studies indicating that some resident microbes may inhibit* H. pylori* growth, specifically Lactobacilli spp. [101, 102]. Discrepancies between studies may be because the ability of* H. pylori* to alter the stomach microbiome is influenced by the species of animal used, genetic background of the animal, specific strain of* H. pylori*, and length of infection. Together these findings indicate that* H. pylori* mediates changes to the host both directly as discussed previously and indirectly by altering the composition and distribution of its natural microbiota.

There are a limited number of studies investigating what effect* H. pylori* has on the microbiome within the human host. One analysis found that the microbial profiles of patients infected with* H. pylori* had increased numbers of non-*Helicobacter* Proteobacteria, Spirochetes, and Acidobacteria as compared to* H. pylori-*negative patients [103]. However, another study examining the effect of* H. pylori* colonization on the gastric microflora showed that* H. pylori* infection causes a shift in the microbiome such that there is an enrichment of Proteobacteria and a decrease in Actinobacteria [104]. Discrepancies in findings may be attributable to variations in bacteria surveillance techniques. As sequencing and analysis technologies improve and become more accessible the perturbation of host flora caused by* H. pylori* colonization will become more clearly defined.

In addition to defining how* H. pylori* alters the composition of the resident microbiome another issue to be resolved is elucidating what impact* H. pylori* mediated dysbiosis has on disease outcome. Specifically, it remains unclear if the gastric microbiota induces a more virulent* H. pylori* or if* H. pylori* induced changes in gastric flora promote carcinogenesis. In the transgenic insulin-gastrin (INS-GAS) mouse model of spontaneous gastric cancer,* H. pylori* drove disease progression and the development of intraepithelial neoplasia. Mice given antibiotics 8 weeks after infection to eradicate* H. pylori* had neoplasia significantly less than mice who received the antibiotics at 12 and 22 weeks after infection. Interestingly,* H. pylori*-free mice given similar antibiotics also displayed a decrease in the development of neoplasia. Taken together these observations indicate that gastric atrophy mediated by* H. pylori* or other factors predisposes to gastric carcinogenesis. The finding that earlier antibiotic treatment was more protective against gastric cancer both in the presence and in the absence of* H. pylori* may be attributable to the eradication of additional, unidentified cancer-potentiating microbes [105]. These findings are further supported by research which showed that germ-free INS-GAS mice had delayed onset of both gastritis and neoplasia compared to specific pathogen-free INS-GAS mice. In the same study* H. pylori*-monocolonization was found to accelerate disease progression resulting in early onset of neoplasia as compared to germ-free mice; however the gastritis was delayed and less severe than* H. pylori* infected mice that maintained a diverse microbiota [106]. The mechanism by which* H. pylori-*associated dysbiosis induces disease progression remains poorly defined. One rationale is that changes in the microbe community include increases of nitrosylating bacterial species which convert nitrogen compounds in gastric fluid to carcinogens such as N-nitrosamines or nitric oxide. Additionally the overgrowth of some bacteria may result in increases in DNA-damaging reactive oxygen species and reactive nitrogen species, which are potent mutagens and can contribute to gastric cancer. Lastly, the dysbiosis created by* H. pylori* may promote host inflammatory responses and accelerate metaplasia, atrophy, and cancer.

## 6. Conclusions

The intersection of host genetics, immune response, bacterial virulence expression, diet, micronutrient availability, and microbiome structure and composition undoubtedly influence the disease outcomes associated with chronic* H. pylori* infection. However, the complex relationship that each of these variables has with each other remains poorly defined. Future studies will seek to determine how these dynamic factors influence each other and can be exploited to ameliorate disease risk and promote gastric health as the age of antibiotics begins to wane.

## References

[1] Moodley Y., Linz B., Bond R. P. (2012). Age of the association between *Helicobacter pylori* and man. *PLoS Pathogens*.

[2] Kodaman N., Pazos A., Schneider B. G. (2014). Human and *Helicobacter pylori* coevolution shapes the risk of gastric disease. *Proceedings of the National Academy of Sciences of the United States of America*.

[3] Kodaman N., Sobota R. S., Mera R., Schneider B. G., Williams S. M. (2014). Disrupted human-pathogen co-evolution: a model for disease. *Frontiers in Genetics*.

[4] Parkin D. M., Bray F., Ferlay J., Pisani P. (2005). Global cancer statistics, 2002. *CA: A Cancer Journal for Clinicians*.

[5] Polk D. B., Peek R. M. (2010). *Helicobacter pylori*: gastric cancer and beyond. *Nature Reviews Cancer*.

[6] Correa P., Piazuelo M. B. (2012). The gastric precancerous cascade. *Journal of Digestive Diseases*.

[7] Schneider B. G., Mera R., Piazuelo M. B. (2015). DNA methylation predicts progression of human gastric lesions. *Cancer Epidemiology Biomarkers & Prevention*.

[8] Wei J., Noto J. M., Zaika E. (2015). Bacterial CagA protein induces degradation of p53 protein in a p14ARF-dependent manner. *Gut*.

[9] Wei J., Nagy T. A., Vilgelm A. (2010). Regulation of p53 tumor suppressor by helicobacter pylori in gastric epithelial cells. *Gastroenterology*.

[10] Hardbower D. M., Peek R. M., Wilson K. T. (2014). At the bench: *Helicobacter pylori*, dysregulated host responses, DNA damage, and gastric cancer. *Journal of Leukocyte Biology*.

[11] Gil R., Sabater-Muñoz B., Latorre A., Silva F. J., Moya A. (2002). Extreme genome reduction in *Buchnera* spp.: toward the minimal genome needed for symbiotic life. *Proceedings of the National Academy of Sciences of the United States of America*.

[12] Dong Q.-J., Wang L.-L., Tian Z.-B., Yu X.-J., Jia S.-J., Xuan S.-Y. (2014). Reduced genome size of *Helicobacter pylori* originating from East Asia. *World Journal of Gastroenterology*.

[13] Fadiel A., Eichenbaum K. D., El Semary N., Epperson B. (2007). Mycoplasma genomics: tailoring the genome for minimal life requirements through reductive evolution. *Frontiers in Bioscience*.

[14] Ghoshal U. C., Tiwari S., Dhingra S. (2008). Frequency of *Helicobacter pylori* and CagA antibody in patients with gastric neoplasms and controls: the Indian enigma. *Digestive Diseases and Sciences*.

[15] Goh K.-L., Cheah P.-L., Md N., Quek K.-F., Parasakthi N. (2007). Ethnicity and *H. pylori* as risk factors for gastric cancer in Malaysia: a prospective case control study. *The American Journal of Gastroenterology*.

[16] Bravo L. E., Van Doorn L.-J., Realpe J. L., Correa P. (2002). Virulence-associated genotypes of Helicobacter pylori: do they explain the African enigma?. *American Journal of Gastroenterology*.

[17] Con S. A., Valerín A. L., Takeuchi H. (2006). Helicobacter pylori CagA status associated with gastric cancer incidence rate variability in Costa Rican regions. *Journal of Gastroenterology*.

[18] Lin D., Koskella B. (2015). Friend and foe: factors influencing the movement of the bacterium *Helicobacter pylori* along the parasitism-mutualism continuum. *Evolutionary Applications*.

[19] Engler D. B., Reuter S., Van Wijck Y. (2014). Effective treatment of allergic airway inflammation with *Helicobacter pylori* immunomodulators requires BATF3-dependent dendritic cells and IL-10. *Proceedings of the National Academy of Sciences of the United States of America*.

[20] Correa P., Cuello C., Duque E. (1976). Gastric cancer in Colombia. III. Natural history of precursor lesions. *Journal of the National Cancer Institute*.

[21] Segal E. D., Lange C., Covacci A., Tompkins L. S., Falkow S. (1997). Induction of host signal transduction pathways by *Helicobacter pylori*. *Proceedings of the National Academy of Sciences of the United States of America*.

[22] Sharma S. A., Tummuru M. K. R., Blaser M. J., Kerr L. D. (1998). Activation of IL-8 gene expression by Helicobacter pylori is regulated by transcription factor nuclear factor-*κ*B in gastric epithelial cells. *Journal of Immunology*.

[23] Tummuru M. K. R., Sharma S. A., Blaser M. J. (1995). *Helicobacter pylori* picB, a homologue of the *Bordetella pertussis* toxin secretion protein, is required for induction of IL-8 in gastric epithelial cells. *Molecular Microbiology*.

[24] Hofman V., Ricci V., Galmiche A. (2000). Effect of Helicobacter pylori on polymorphonuclear leukocyte migration across polarized T84 epithelial cell monolayers: role of vacuolating toxin VacA and cag pathogenicity island. *Infection and Immunity*.

[25] Su B., Ceponis P. J. M., Sherman P. M. (2003). Cytoskeletal rearrangements in gastric epithelial cells in response to *Helicobacter pylori* infection. *Journal of Medical Microbiology*.

[26] McClain M. S., Iwamoto H., Cao P. (2003). Essential role of a GXXXG motif for membrane channel formation by *Helicobacter pylori* vacuolating toxin. *The Journal of Biological Chemistry*.

[27] Cover T. L., Blanke S. R. (2005). *Helicobacter pylori* VacA, a paradigm for toxin multifunctionality. *Nature Reviews Microbiology*.

[28] Cover T. L., Vaughn S. G., Cao P., Blaser M. J. (1992). Potentiation of *Helicobacter pylori* vacuolating toxin activity by nicotine and other weak bases. *Journal of Infectious Diseases*.

[29] Merrell D. S., Thompson L. J., Kim C. C. (2003). Growth phase-dependent response of *Helicobacter pylori* to iron starvation. *Infection and Immunity*.

[30] Moonens K., Gideonsson P., Subedi S. (2016). Structural insights into polymorphic ABO glycan binding by *Helicobacter pylori*. *Cell Host & Microbe*.

[31] Ishijima N., Suzuki M., Ashida H. (2011). BabA-mediated adherence is a potentiator of the *Helicobacter pylori* type IV secretion system activity. *The Journal of Biological Chemistry*.

[32] Mahdavi J., Sondén B., Hurtig M. (2002). *Helicobacter pylori* SabA adhesin in persistent infection and chronic inflammation. *Science*.

[33] Peck B., Ortkamp M., Diehl K. D., Hundt E., Knapp B. (1999). Conservation, localization and expression of HopZ, a protein involved in adhesion of *Helicobacter pylori*. *Nucleic Acids Research*.

[34] Giannakis M., Bäckhed H., Chen S. L. (2009). Response of gastric epithelial progenitors to *Helicobacter pylori* isolates obtained from Swedish patients with chronic atrophic gastritis. *The Journal of Biological Chemistry*.

[35] Franco A. T., Johnston E., Krishna U. (2008). Regulation of gastric carcinogenesis by *Helicobacter pylori* virulence factors. *Cancer Research*.

[36] Aspholm M., Olfat F. O., Nordén J. (2006). SabA is the H. pylori hemagglutinin and is polymorphic in binding to sialylated glycans. *PLoS Pathogens*.

[37] Setiawan V. W., Zhang Z.-F., Yu G.-P. (2001). Protective effect of green tea on the risks of chronic gastritis and stomach cancer. *International Journal of Cancer*.

[38] Lunet N., Lacerda-Vieira A., Barros H. (2005). Fruit and vegetables consumption and gastric cancer: a systematic review and meta-analysis of cohort studies. *Nutrition and Cancer*.

[39] Takezaki T., Gao C.-M., Wu J.-Z. (2001). Dietary protective and risk factors for esophageal and stomach cancers in a low-epidemic area for stomach cancer in Jiangsu Province, China: comparison with those in a high-epidemic area. *Japanese Journal of Cancer Research*.

[40] Joossens J. V., Hill M. J., Elliott P. (1996). Dietary salt, nitrate and stomach cancer mortality in 24 countries. European Cancer Prevention (ECP) and the INTERSALT Cooperative Research Group. *International Journal of Epidemiology*.

[41] Gonzalez C. A., Riboli E. (2006). Diet and cancer prevention: where we are, where we are going. *Nutrition and Cancer*.

[42] Pakseresht M., Forman D., Malekzadeh R. (2011). Dietary habits and gastric cancer risk in north-west Iran. *Cancer Causes and Control*.

[43] Nouraie M., Pietinen P., Kamangar F. (2005). Fruits, vegetables, and antioxidants and risk of gastric cancer among male smokers. *Cancer Epidemiology Biomarkers and Prevention*.

[44] Botterweck A. A. M., van den Brandt P. A., Goldbohm R. A. (2000). Vitamins, carotenoids, dietary fiber, and the risk of gastric carcinoma: results from a prospective study after 6.3 years of follow-up. *Cancer*.

[45] D'Elia L., Rossi G., Ippolito R., Cappuccio F. P., Strazzullo P. (2012). Habitual salt intake and risk of gastric cancer: a meta-analysis of prospective studies. *Clinical Nutrition*.

[46] Kneller R. W., Guo W.-D., Hsing A. W. (1992). Risk factors for stomach cancer in sixty-five Chinese counties. *Cancer Epidemiology Biomarkers and Prevention*.

[47] Fox J. G., Rogers A. B., Ihrig M. (2003). *Helicobacter pylori*-associated gastric cancer in INS-GAS mice is gender specific. *Cancer Research*.

[48] Rogers A. B., Taylor N. S., Whary M. T., Stefanich E. D., Wang T. C., Fox J. G. (2005). Helicobacter pylori but not high salt induces gastric intraepithelial neoplasia in B6129 mice. *Cancer Research*.

[49] Fox J. G., Dangler C. A., Taylor N. S., King A., Koh T. J., Wang T. C. (1999). High-salt diet induces gastric epithelial hyperplasia and parietal cell loss, and enhances *Helicobacter pylori* colonization in C57BL/6 mice. *Cancer Research*.

[50] Gaddy J. A., Radin J. N., Loh J. T. (2013). High dietary salt intake exacerbates *Helicobacter pylori*-induced gastric carcinogenesis. *Infection and Immunity*.

[51] Loh J. T., Torres V. J., Cover T. L. (2007). Regulation of *Helicobacter pylori* cagA expression in response to salt. *Cancer Research*.

[52] Voss B. J., Loh J. T., Hill S., Rose K. L., Mcdonald W. H., Cover T. L. (2015). Alteration of the *Helicobacter pylori* membrane proteome in response to changes in environmental salt concentration. *Proteomics—Clinical Applications*.

[53] Testerman T. L., Conn P. B., Mobley H. L. T., McGee D. J. (2006). Nutritional requirements and antibiotic resistance patterns of Helicobacter species in chemically defined media. *Journal of Clinical Microbiology*.

[54] Choby J. E., Skaar E. P. (2016). Heme synthesis and acquisition in bacterial pathogens. *Journal of Molecular Biology*.

[55] Skaar E. P., Raffatellu M. (2015). Metals in infectious diseases and nutritional immunity. *Metallomics*.

[56] Haley K. P., Skaar E. P. (2012). A battle for iron: host sequestration and *Staphylococcus aureus* acquisition. *Microbes and Infection*.

[57] Morgenthau A., Pogoutse A., Adamiak P., Moraes T. F., Schryvers A. B. (2013). Bacterial receptors for host transferrin and lactoferrin: molecular mechanisms and role in host-microbe interactions. *Future Microbiology*.

[58] Ganz T. (2003). Hepcidin, a key regulator of iron metabolism and mediator of anemia of inflammation. *Blood*.

[59] Senkovich O., Ceaser S., McGee D. J., Testerman T. L. (2010). Unique host iron utilization mechanisms of Helicobacter pylori revealed with iron-deficient chemically defined media. *Infection and Immunity*.

[60] Danielli A., Romagnoli S., Roncarati D., Costantino L., Delany I., Scarlato V. (2009). Growth phase and metal-dependent transcriptional regulation of the fecA genes in *Helicobacter pylori*. *Journal of Bacteriology*.

[61] Ernst F. D., Bereswill S., Waidner B. (2005). Transcriptional profiling of *Helicobacter pylori* Fur- and iron-regulated gene expression. *Microbiology*.

[62] Delany I., Pacheco A. B. F., Spohn G., Rappuoli R., Scarlato V. (2001). Iron-dependent transcription of the frpB gene of *Helicobacter pylori* is controlled by the Fur repressor protein. *Journal of Bacteriology*.

[63] Pich O. Q., Carpenter B. M., Gilbreath J. J., Merrell D. S. (2012). Detailed analysis of Helicobacter pylori Fur-regulated promoters reveals a Fur box core sequence and novel Fur-regulated genes. *Molecular Microbiology*.

[64] Vannini A., Roncarati D., Spinsanti M., Scarlato V., Danielli A. (2014). In depth analysis of the *Helicobacter pylori* cag pathogenicity island transcriptional responses. *PLoS ONE*.

[65] Haley K. P., Blanz E. J., Gaddy J. A. (2014). High resolution electron microscopy of the Helicobacter pylori cag type IV secretion system pili produced in varying conditions of iron availability. *Journal of Visualized Experiments*.

[66] Noto J. M., Gaddy J. A., Lee J. Y. (2013). Iron deficiency accelerates *Helicobacter pylori*-induced carcinogenesis in rodents and humans. *The Journal of Clinical Investigation*.

[67] Tan S., Noto J. M., Romero-Gallo J., Peek R. M., Amieva M. R. (2011). *Helicobacter pylori* perturbs iron trafficking in the epithelium to grow on the cell surface. *PLoS Pathogens*.

[68] Yuan Y., Wu Q., Cheng G. (2015). Recombinant human lactoferrin enhances the efficacy of triple therapy in mice infected with *Helicobacter pylori*. *International Journal of Molecular Medicine*.

[69] Akiba S., Neriishi K., Blot W. J. (1991). Serum ferritin and stomach cancer risk among a Japanese population. *Cancer*.

[70] Nomura A., Chyou P.-H., Stemmermann G. N. (1992). Association of serum ferritin levels with the risk of stomach cancer. *Cancer Epidemiology Biomarkers and Prevention*.

[71] Harrison L. E., Zhang Z.-F., Karpeh M. S., Sun M., Kurtz R. C. (1997). The role of dietary factors in the intestinal and diffuse histologic subtypes of gastric adenocarcinoma: a Case-Control Study in the U.S.. *Cancer*.

[72] Haley K. P., Delgado A. G., Piazuelo M. B. (2015). The human antimicrobial protein calgranulin C participates in control of *Helicobacter pylori* growth and regulation of virulence. *Infection and Immunity*.

[73] Gaddy J. A., Radin J. N., Loh J. T. (2014). The host protein calprotectin modulates the *Helicobacter pylori* cag Type IV secretion system via zinc sequestration. *PLoS Pathogens*.

[74] Sempértegui F., Díaz M., Mejía R. (2007). Low concentrations of zinc in gastric mucosa are associated with increased severity of *Helicobacter pylori*-induced inflammation. *Helicobacter*.

[75] Gaddy J. A., Radin J. N., Cullen T. W. (2015). *Helicobacter pylori* resists the antimicrobial activity of calprotectin via lipid A modification and associated biofilm formation. *mBio*.

[76] Dawsey S. P., Hollenbeck A., Schatzkin A., Abnet C. C. (2014). A prospective study of vitamin and mineral supplement use and the risk of upper gastrointestinal cancers. *PLoS ONE*.

[77] Toyonaga A., Okamatsu H., Sasaki K. (2000). Epidemiological study on food intake and *Helicobacter pylori* infection. *Kurume Medical Journal*.

[78] Janjetic M. A., Goldman C. G., Balcarce N. E. (2010). Iron, zinc, and copper nutritional status in children infected with *Helicobacter pylori*. *Journal of Pediatric Gastroenterology and Nutrition*.

[79] Baik S. J., Yi S. Y., Park H. S., Park B. H. (2012). Seroprevalence of *Helicobacter pylori* in female Vietnamese immigrants to Korea. *World Journal of Gastroenterology*.

[80] Herrmann L., Schwan D., Garner R. (1999). Helicobacter pylori cadA encodes an essential Cd(II)-Zn(II)-Co(II) resistance factor influencing urease activity. *Molecular Microbiology*.

[81] Zambelli B., Turano P., Musiani F., Neyroz P., Ciurli S. (2009). Zn^2+^-linked dimerization of UreG from *Helicobacter pylori*, a chaperone involved in nickel trafficking and urease activation. *Proteins: Structure, Function and Bioinformatics*.

[82] Sydor A. M., Lebrette H., Ariyakumaran R., Cavazza C., Zamble D. B. (2014). Relationship between Ni(II) and Zn(II) coordination and nucleotide binding by the helicobacter pylori [NiFe]-hydrogenase and urease maturation factor HypB. *Journal of Biological Chemistry*.

[83] Bellucci M., Zambelli B., Musiani F., Turano P., Ciurli S. (2009). *Helicobacter pylori* UreE, a urease accessory protein: Specific Ni^2+^- and Zn^2+^-binding properties and interaction with its cognate UreG. *Biochemical Journal*.

[84] Johnson R. C., Hu H. Q., Merrell D. S., Maroney M. J. (2015). Dynamic HypA zinc site is essential for acid viability and proper urease maturation in *Helicobacter pylori*. *Metallomics*.

[85] Xia W., Li H., Sze K.-H., Sun H. (2009). Structure of a nickel chaperone, HypA, from *Helicobacter pylori* reveals two distinct metal binding sites. *Journal of the American Chemical Society*.

[86] Herbst R. W., Perovic I., Martin-Diaconescu V. (2010). Communication between the zinc and nickel sites in dimeric HypA: metal recognition and pH sensing. *Journal of the American Chemical Society*.

[87] Waidner B., Melchers K., Stähler F. N., Kist M., Bereswill S. (2005). The *Helicobacter pylori* CrdRS two-component regulation system (HP1364/HP1365) is required for copper-mediated induction of the copper resistance determinant CrdA. *Journal of Bacteriology*.

[88] Waidner B., Melchers K., Ivanov I. (2002). Identification by RNA profiling and mutational analysis of the novel copper resistance determinants CrdA (HP1326), CrdB (HP1327), and CzcB (HP1328) in *Helicobacter pylori*. *Journal of Bacteriology*.

[89] Stähler F. N., Odenbreit S., Haas R. (2006). The novel *Helicobacter pylori* CznABC metal efflux pump is required for cadmium, zinc, and nickel resistance, urease modulation, and gastric colonization. *Infection and Immunity*.

[90] Tran C. D., Campbell M. A. F., Kolev Y., Chamberlain S., Huynh H. Q., Butler R. N. (2005). Short-term zinc supplementation attenuates *Helicobacter felis*-induced gastritis in the mouse. *Journal of Infection*.

[91] Picot J., Hartwell D., Harris P., Mendes D., Clegg A. J., Takeda A. (2012). The effectiveness of interventions to treat severe acute malnutrition in young children: a systematic review. *Health Technology Assessment*.

[92] Campanale M., Nucera E., Ojetti V. (2014). Nickel free-diet enhances the Helicobacter pylori eradication rate: a pilot study. *Digestive Diseases and Sciences*.

[93] MacOmber L., Hausinger R. P. (2011). Mechanisms of nickel toxicity in microorganisms. *Metallomics*.

[94] de Reuse H., Vinella D., Cavazza C. (2013). Common themes and unique proteins for the uptake and trafficking of nickel, a metal essential for the virulence of *Helicobacter pylori*. *Frontiers in Cellular and Infection Microbiology*.

[95] Fulkerson J. F., Mobley H. L. (2000). Membrane topology of the NixA nickel transporter of *Helicobacter pylori*: two nickel transport-specific motifs within transmembrane helices II and III. *Journal of Bacteriology*.

[96] Schauer K., Gouget B., Carrière M., Labigne A., De Reuse H. (2007). Novel nickel transport mechanism across the bacterial outer membrane energized by the TonB/ExbB/ExbD machinery. *Molecular Microbiology*.

[97] Stoof J., Kuipers E. J., van Vliet A. H. M. (2010). Characterization of NikR-responsive promoters of urease and metal transport genes of *Helicobacter mustelae*. *BioMetals*.

[98] Stoof J., Kuipers E. J., Klaver G., Van Vliet A. H. M. (2010). An ABC transporter and a TonB ortholog contribute to Helicobacter mustelae nickel and cobalt acquisition. *Infection and Immunity*.

[99] Aebischer T., Fischer A., Walduck A. (2006). Vaccination prevents *Helicobacter pylori*-induced alterations of the gastric flora in mice. *FEMS Immunology and Medical Microbiology*.

[100] Yin Y.-N., Wang C.-L., Liu X.-W. (2011). Gastric and duodenum microflora analysis after long-term *Helicobacter pylori* infection in Mongolian gerbils. *Helicobacter*.

[101] Osaki T., Matsuki T., Asahara T. (2012). Comparative analysis of gastric bacterial microbiota in Mongolian gerbils after long-term infection with *Helicobacter pylori*. *Microbial Pathogenesis*.

[102] Sun Y.-Q., Monstein H.-J., Nilsson L. E., Petersson F., Borch K. (2003). Profiling and identification of eubacteria in the stomach of Mongolian gerbils with and without *Helicobacter pylori* infection. *Helicobacter*.

[103] Maldonado-Contreras A., Goldfarb K. C., Godoy-Vitorino F. (2011). Structure of the human gastric bacterial community in relation to *Helicobacter pylori* status. *ISME Journal*.

[104] Aviles-Jimenez F., Vazquez-Jimenez F., Medrano-Guzman R., Mantilla A., Torres J. (2014). Stomach microbiota composition varies between patients with non-atrophic gastritis and patients with intestinal type of gastric cancer. *Scientific Reports*.

[105] Lee C.-W., Rickman B., Rogers A. B., Ge Z., Wang T. C., Fox J. G. (2008). Helicobacter pylori eradication prevents progression of gastric cancer in hypergastrinemic INS-GAS mice. *Cancer Research*.

[106] Lofgren J. L., Whary M. T., Ge Z. (2011). Lack of commensal flora in helicobacter pylori-infected INS-GAS mice reduces gastritis and delays intraepithelial neoplasia. *Gastroenterology*.

